# Accurately predicting electron beam deflections in fringing fields of a solenoid

**DOI:** 10.1038/s41598-020-67596-0

**Published:** 2020-07-02

**Authors:** Christof Baumgärtel, Ray T. Smith, Simon Maher

**Affiliations:** Department of Electrical Engineering and Electronics, University of Liverpool, Liverpool, UK

**Keywords:** Electrical and electronic engineering, Techniques and instrumentation, Physics

## Abstract

Computer modelling is widely used in the design of scientific instrumentation for manipulating charged particles, for instance: to evaluate the behaviour of proposed designs, to determine the effects of manufacturing imperfections and to optimise the performance of apparatus. For solenoids, to predict charged particle trajectories, accurate values for the magnetic field through which charged species traverse are required, particularly at the end regions where fringe fields are most prevalent. In this paper, we describe a model that accurately predicts the deflection of an electron beam trajectory in the vicinity of the fringing field of a solenoid. The approach produces accurate beam deflection predictions in the fringe field region as well as in the centre of the solenoid. The model is based on a direct-line-of-action force between charges and is compared against field-based approaches including a commercially available package, with experimental verification (for three distinct cases). The direct-action model is shown to be more accurate than the other models relative to the experimental results obtained.

## Introduction

Charged particle dynamics (also known as charged particle optics) is concerned with the manipulation of charged particles (e.g., electrons, protons, ions) within an electric and/or magnetic field. The deflection of charged particles by electromagnetic fields has widespread and important connotations in physical sciences, engineering, life sciences and for a wide range of technologies. An understanding of the electrical forces at play between charges is essential and has long been applied to a multitude of high-tech applications, from particle accelerators^1–4^, decelerators (e.g., storage rings)^5,6^, electron microscopes^7,8^, electronics ^9^, nuclear fusion reactors^10,11^ and magnetrons^12,13^, through to medical diagnostics (e.g., radiation therapy)^14,15^, mass spectrometry^16^ and electron beam welding^17,18^, amongst many others.

The simulation and prediction of beam trajectories is of great importance for design purposes, especially for low energy beams where perturbing effects such as space charge and fringe fields are prevalent in the low velocity regime and can impede the accuracy of predictions. In particular, fringe fields are known to introduce non-linearities that induce a variety of undesired effects, such as influencing particle motion by sudden shifts in position, as well as tune shifts and chromaticity shifts^19^ leading to beam instability. When developing new instrumentation for manipulating charged species, both the cost and practicality of assembly make it extremely difficult and time-consuming to test (experimentally) the behaviour of different designs. Hence, it is common for many of the application areas already noted to utilise accurate simulation of performance prior to construction. This is an important step when designing charged particle apparatus and particularly for more advanced systems, such as unconventional and/or high performance setups, as it is vital in securing cost-effectiveness and functionality.

Solutions for electromagnetic problems, including those encountered in the field of charged particle optics, are often solved numerically using the finite element (FE)^20^, finite difference (FD)^21^ or boundary element (BE) methods^22^. These methods have been hugely successful and each has its own benefits and drawbacks broadly relating to accuracy, resolution, computational time and memory requirements^23^, yet fundamentally they are all solving the same equation set based on Maxwell–Lorentz.

In this paper we present a novel model for charged particle deflection in the low velocity regime, $v \ll c$, which is formed from a fundamentally different approach as compared to the field theory approach of Maxwell–Lorentz. According to field theory, a particle’s deflection is governed by the Lorentz force equation,

1 $$\begin{aligned} \vec {F} = q (\vec {E} + \vec {v} \times \vec {B}), \end{aligned}$$

that relates the deflection of a charge, *q*, moving at speed, $\vec {v}$, to the electrical field strength, $\vec {E}$, and the magnetic field strength, $\vec {B}$. By contrast, the new model proposed herein, is based on a direct physical approach involving a direct-line-of-action force between charges in the low velocity regime. Specifically, we are concerned with the interaction between the charge carriers in an electron beam and those within a current carrying solenoid, which forms the basis of an investigation to predict the behaviour of a charged particle beam in the region of a fringing magnetic field. The solenoid is key in this regard, as it has long been established as a fundamental charge optical element for generating a controlled magnetic field that is widely used for manipulating charged particles (e.g., focusing and beam guiding). Moreover, it is well-known that the motion of charged particles in the vicinity of a solenoid, particularly near the entrance and exit, are subject to non-linear effects that can have a significant impact on particle trajectories that are difficult to accurately predict^24–27^.

Our model is compared with the predictions of field-theory, including state-of-the-art commercially available software^22^ as well as calculations by Cébron^28^ which are based on the work of Callaghan and Maslen^29^ and Derby and Olbert^30^. These field calculations form the basis of further work on finite solenoids with a permeable core^31^, internal magnetic fields of off-axis solenoids^32^ and parallel finite solenoids^33^, even to the application of axis alignment measurements of solenoids^34–36^.

To evaluate the validity and accuracy of both model types (field versus direct-line-of-action force), an experiment is set up that employs a modified electron gun whose beam is made to pass across a variety of current carrying solenoids from whence the beam is deflected. By locating the solenoids at different positions with respect to the electron gun, it is then possible to investigate the deflection caused by significant fringing fields which are associated with short solenoids and the end regions of a given solenoid.

## Mathematical model

Three different models which predict the deflection of an electron beam passing across a solenoid in any field region are presented and explained in this section: (i) the standard (i.e., conventional) model following the field-based approach of electrodynamics (Sect. ‘Field model’) where the interaction of all charged particles is based on the Maxwellian theory of electric and magnetic fields^28–30^; (ii) a numerical model based on field theory (Sect. ‘Modelling with CPO’) from the software package CPO^22^, which is used to calculate beam trajectories based on the Boundary-Element-Method (BEM); (iii) our model (Sect. ‘Weber model’) which employs the theory of direct action at a distance between the particles and is based on Weber electrodynamics, where the concept of field entities $\vec {E}$ & $\vec {B}$ and their mediation is avoided. In this paper, we develop a new model based on the Weber force equation to predict the trajectory of an electron beam and its deflection accordingly. Recently, there has been increased research interest in Weber-based electrodynamics, where the theory has been related not only to electron beam deflection^37,38^, but also to induction^39,40^, superconductivity^41,42^ as well as the Planck-constant^43,44^ and the structure of the atom^45,46^. First, the Weber-based model will be explained in the following section.

### Weber model

The model we present follows the direct action approach laid out by Weber^47^, where he published his findings 15 years prior to Maxwell’s theory^48^ of fields and aether. The interaction of charged particles according to Weber is determined by the Weber-force equation,

2 $$\begin{aligned} \vec {F}_{21} = \frac{q_1 q_2}{4 \pi \varepsilon _0} \frac{\hat{r}_{12}}{r_{12}^2} \left( 1 - \frac{\dot{r}_{12}^2}{2 c^2} + \frac{r_{12} \ddot{r}_{12}}{c^2} \right) \end{aligned}$$

that depends on the relative displacement $\vec {r_{12}}$, relative velocity $\dot{r_{12}}$ and relative acceleration $\ddot{r_{12}}$ between the charges $q_1$ and $q_2$, where $\varepsilon _0$ is the permittivity of free space and *c* is the speed of light. In this formulation we have

3 $$\begin{aligned} \vec {r}_{12}= & {} \vec {r}_1 - \vec {r}_2 \end{aligned}$$

4 $$\begin{aligned} \dot{r}_{12}= & {} \frac{dr_{12}}{dt} = \hat{r}_{12} \cdot \vec {v}_{12} \end{aligned}$$

5 $$\begin{aligned} \ddot{r}_{12}= & {} \frac{d^2r_{12}}{dt^2} = \frac{d \dot{r}_{12}}{dt} = \frac{[ \vec {v}_{12} \cdot \vec {v}_{12} - (\hat{r}_{12} \cdot \vec {v}_{12})^2 + \vec {r}_{12} \cdot \vec {a}_{12}]}{r_{12}} \end{aligned}$$

along with the magnitude and unit vector in equation (6).

6 $$\begin{aligned} r_{12} = | \vec {r}_1 - \vec {r}_2 | \qquad \hat{r}_{12} = \frac{\vec {r}_{12}}{r_{12}} \end{aligned}$$

**Figure 1:**
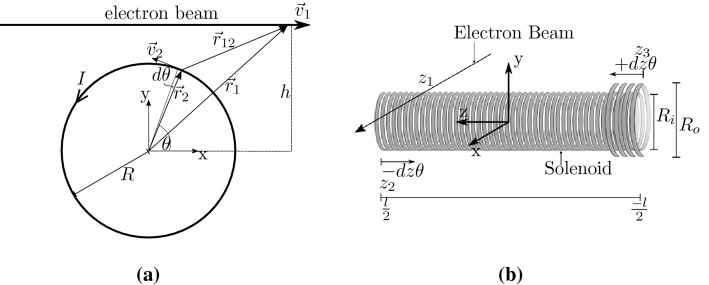
Geometry and system of coordinates to model the electron beam and solenoid: **a** Simplified 2D geometry for a single current carrying loop and an electron beam travelling parallel to the x-axis; **b** Expanded 3D geometry with the coordinate origin at the centre of the double wound solenoid with the beam at a position, $z_1$, travelling parallel to the x-axis.

The Weber force acts along the line joining the particles and reduces to the Coulomb force for charges at rest. It is also consistent with the field equations of Maxwell^49–52^, although it does not depend on them conceptually, as it is a direct-action approach where a form of mediation is not needed to transmit the force between charges. Based on this force approach a model has been derived in an earlier work^37^ for the deflection of electrons across a single current loop, but to apply this to the fringing field regions, significant refinements have been added to the simplified two dimensional (2D) model of^37^, especially to account for the three dimensional (3D) nature, taking the geometry of the particular solenoid into account. As a first step, however, a single current loop can be assumed in a 2D plane, where the electron beam is traversing across the solenoid at a fixed height, h, in the positive x-direction with a velocity, $\vec {v}_1$, giving it a position, $\vec {r}_1$, as shown in Fig. 1a.

The current, *I*, is situated at a position, $\vec {r}_2$, and rotating here in a positive mathematical sense, with a velocity, $\vec {v}_2$, around the loop with a radius, *R*, so that it can be represented by a current element, $IR d\theta$. It is important to note that the rotational direction of the current is regarded as the actual physical motion undertaken by the electrons in the current element, so it is in opposition to the conventional current flow, and rather sticks to the physical direction of current flow.

As a next step the model is now extended to 3D space, with the coordinate system being centred in the solenoid, with the solenoid axis elongating from $-\frac{l}{2}$ to $\frac{l}{2}$. For the experiments, the solenoids used are all double wound with a given number of windings, and to take the double wound manufacturing into account in the model, the force is split into an inner and outer component, $F_i$ and $F_o$, respectively, with a coil radius of $R_i$ and $R_o$ which are about the same, $R_i \approx R_o$. We assume that the electron-current is fed to the solenoid at the position $z_2 = \frac{l}{2}$ to one layer of the winding, so that it moves towards $z_3 = -\frac{l}{2}$ and is then returning through the other layer towards $z_2$, as depicted in Fig. 1b.

This behaviour can be modelled in two ways, one is a helical motion following the pitch of the windings and the other one is a summation model, where each winding is assumed as a single current loop at a given position and the principle of superposition is used to summate the force contribution from each individual loop. For the helical model the current is then changing its axial position, depending on the pitch with $dz \cdot \theta$, either in a positive or negative sense, depending on where the current is fed and returned to. The corresponding velocity increase along the axis is derived as $\frac{v_2}{R} dz$ and the relational position, $\vec {r}_{12}$, and velocity, $\vec {v}_{12}$, are defined as:

7 $$\begin{aligned} \vec {r}_1&= \begin{pmatrix} x_1 \\ h \\ z_1 \end{pmatrix} \quad \vec {r}_{2i} = \begin{pmatrix} R \cos (\theta ) \\ R \sin (\theta ) \\ z_2-dz \theta \end{pmatrix} \quad \vec {r}_{2o} = \begin{pmatrix} R \cos (\theta ) \\ R \sin (\theta ) \\ z_3+dz \theta \end{pmatrix} \end{aligned}$$

8 $$\begin{aligned} \vec {r}_{12i}&= \begin{pmatrix} x_1-R \cos (\theta ) \\ h-R \sin (\theta ) \\ z_1-z_2 + dz \theta \end{pmatrix} \vec {r}_{12o} = \begin{pmatrix} x_1-R \cos (\theta ) \\ h-R \sin (\theta ) \\ z_1-z_3 - dz \theta \end{pmatrix} \end{aligned}$$

9 $$\begin{aligned} r_{12i}&= \sqrt{(x_1-R \cos (\theta ))^2 + (h-R \sin (\theta ))^2 + (z_1-z_2 + dz \theta )^2} \end{aligned}$$

10 $$\begin{aligned} r_{12o}&= \sqrt{(x_1-R \cos (\theta ))^2 + (h-R \sin (\theta ))^2 + (z_1-z_3 - dz \theta )^2} \end{aligned}$$

11 $$\begin{aligned} \vec {v}_1&= \begin{pmatrix} v_1 \\ 0 \\ 0 \end{pmatrix} \quad \vec {v}_{2i} = \begin{pmatrix} -v_2 \sin (\theta ) \\ v_2 \cos (\theta ) \\ -\frac{v_2}{R}dz \end{pmatrix} \quad \vec {v}_{2o} = \begin{pmatrix} -v_2 \sin (\theta ) \\ v_2 \cos (\theta ) \\ \frac{v_2}{R}dz \end{pmatrix} \quad \vec {v}_{12_{i,o}} = \begin{pmatrix} v_1 + v_2 \sin (\theta ) \\ - v_2 \cos (\theta ) \\ \pm \frac{v_2}{R}dz \end{pmatrix} \end{aligned}$$

It is further assumed that the acceleration is negligible for the given experimental setup, as the electrons in the beam are moving at a constant speed after the initial acceleration phase and the acceleration that is exerted on the current element due to the helical motion is sufficiently small.

In order to calculate the resulting force between the beam and the current in the solenoid a superposition of all the charges involved must be performed. Physically, the electrons in the beam are interacting with the electrons in the current element and also the positive charges in the static metallic lattice of the solenoid. With $$q_{1-} = -q_{1+}$$ and $$q_{2-} = - q_{2+} $$, as well as $$v_1 \gg v_2$$, we get:

12 $$\begin{aligned} \vec {F}_{i_{helix}}&= \vec {F}_{i_{1-2-}} + \vec {F}_{i_{1-2+}} \nonumber \\&=\frac{q_{1+} q_{2+}}{4 \pi \varepsilon _0} \frac{v_1 v_2}{c^2} \frac{\vec {r}_{12i}}{r_{12i}^3} \left\{ 2 \sin \theta - 3 \sin \theta \frac{(x-R \cos \theta )^2}{r_{12i}^2} + 3 \cos \theta \frac{(x-R \cos \theta )(h-R \sin \theta )}{r_{12i}^2} \right. \nonumber \\&\qquad \qquad \left. - 3 \frac{dz}{R} \frac{(x-R \cos \theta )(z_1-z_2+dz \theta )}{r_{12i}^2} \right\} \end{aligned}$$

13 $$\begin{aligned} \vec {F}_{o_{helix}}&= \vec {F}_{o_{1-2-}} + \vec {F}_{o_{1-2+}} \nonumber \\&= \frac{q_{1+} q_{2+}}{4 \pi \varepsilon _0} \frac{v_1 v_2}{c^2} \frac{\vec {r}_{12o}}{r_{12o}^3} \left\{ 2 \sin \theta - 3 \sin \theta \frac{(x-R \cos \theta )^2}{r_{12o}^2} + 3 \cos \theta \frac{(x-R \cos \theta )(h-R \sin \theta )}{r_{12o}^2} \right. \nonumber \\&\qquad \qquad \left. + 3 \frac{dz}{R} \frac{(x-R \cos \theta )(z_1-z_3-dz \theta )}{r_{12o}^2} \right\} \end{aligned}$$

For the summation model each single loop is assumed to be positioned at one distinct point on the solenoid axis $p \cdot (n-1)$, with *p* being the pitch of the winding and the integer index $n = 1 \dots N$, where *N* is the total number of turns in one layer. Accordingly, the current element is only moving in the 2D plane at each loop, thus not having an axial velocity component. The inner and outer forces are then found to be:

14 $$\begin{aligned} \vec {F}_{i_{summation}}&= \vec {F}_{i_{1-2-}} + \vec {F}_{i_{1-2+}} \nonumber \\&= \frac{q_{1+} q_{2+}}{4 \pi \varepsilon _0} \frac{v_1 v_2}{c^2} \frac{\vec {r}_{12i}}{r_{12i}^3} \left\{ 2 \sin \theta - 3 \sin \theta \frac{(x-R \cos \theta )^2}{r_{12i}^2} + 3 \cos \theta \frac{(x-R \cos \theta )(h-R \sin \theta )}{r_{12i}^2} \right\} \end{aligned}$$

15 $$\begin{aligned} \vec {F}_{o_{summation}}&= \vec {F}_{o_{1-2-}} + \vec {F}_{o_{1-2+}} \nonumber \\&= \frac{q_{1+} q_{2+}}{4 \pi \varepsilon _0} \frac{v_1 v_2}{c^2} \frac{\vec {r}_{12o}}{r_{12o}^3} \left\{ 2 \sin \theta - 3 \sin \theta \frac{(x-R \cos \theta )^2}{r_{12o}^2} + 3 \cos \theta \frac{(x-R \cos \theta )(h-R \sin \theta )}{r_{12o}^2} \right\} \end{aligned}$$

To evaluate the total force exerted by the solenoid on the beam, we can assume that while the continuous current is flowing, a current element is immediately replaced with the next. The transformation $qv \rightarrow \lambda v \rightarrow I R d\theta$ then holds the total force for integrating over the complete solenoid. This yields force equations for both Weber-based models,

16 $$\begin{aligned} \vec {F}_{W_{helix}}&= \frac{q_{1+} v_1 I}{4 \pi \varepsilon _0 c^2} \int _{0}^{N \cdot 2 \pi } \left\{ \frac{\vec {r}_{12i}}{r_{12i}^3} \left[ 2 \sin \theta - 3 \sin \theta \frac{(x-R \cos \theta )^2}{r_{12i}^2} + 3 \cos \theta \frac{(x-R \cos \theta )(h-R \sin \theta )}{r_{12i}^2} \right. \right. \nonumber \\&\qquad \qquad \left. -\, 3 \frac{dz}{R} \frac{(x-R \cos \theta )(z_1-z_2+dz \theta )}{r_{12i}^2} \right] + \frac{\vec {r}_{12o}}{r_{12o}^3} \left[ 2 \sin \theta - 3 \sin \theta \frac{(x-R \cos \theta )^2}{r_{12o}^2} \right. \nonumber \\&\qquad \qquad \left. \left. +\, 3 \cos \theta \frac{(x-R \cos \theta )(h-R \sin \theta )}{r_{12o}^2} + 3 \frac{dz}{R} \frac{(x-R \cos \theta )(z_1-z_3-dz \theta )}{r_{12o}^2} \right] \right\} R d \theta \end{aligned}$$

17 $$\begin{aligned} \vec {F}_{W_{sum}}&= \frac{q_{1+} v_1 I}{4 \pi \varepsilon _0 c^2} \sum _{n = 1}^{N} \int _{0}^{2 \pi } \left\{ \frac{\vec {r}_{12i}}{r_{12i}^3} \left[ 2 \sin \theta - 3 \sin \theta \frac{(x-R \cos \theta )^2}{r_{12i}^2} + 3 \cos \theta \frac{(x-R \cos \theta )(h-R \sin \theta )}{r_{12i}^2} \right. \right. \nonumber \\&\quad + \frac{\vec {r}_{12o}}{r_{12o}^3} \left[ 2 \sin \theta - 3 \sin \theta \frac{(x-R \cos \theta )^2}{r_{12o}^2} \left. + 3 \cos \theta \frac{(x-R \cos \theta )(h-R \sin \theta )}{r_{12o}^2} \right] \right\} R d \theta \end{aligned}$$

where *N* is the number of turns in one layer. From both of these equations it is evident that the model predicts three kinds of forces on the beam: a longitudinal force, a vertically transversal force and a horizontally transversal force. The beam will then be deflected in the y-direction due to the transverse (vertical) force and in the z-direction due to the (horizontal) transverse force. Therefore the impulse of the force is calculated with the time, *t*, that the beam spends travelling from the anode to a fluorescent screen (that is excited upon electron collision) on which the deflection is recorded.

18 $$\begin{aligned} \vec {J} = \int _{0}^{t} \vec {F}_W dt \end{aligned}$$

Equating this to the change of vertical and horizontal momentum,

19 $$\begin{aligned} J_v = m_e v_v, \qquad J_h = m_e v_h \end{aligned}$$

where $m_e$ is the electron mass and $v_h,~v_v$ are the horizontal and vertical velocities gained from the deflection force, the total deflections can be calculated as,

20 $$\begin{aligned} y_d = \frac{1}{2} a_v t^2 = \frac{1}{2} v_v t = \frac{1}{2} \frac{J_v}{m_e} t, \qquad z_d = \frac{1}{2} a_h t^2 = \frac{1}{2} v_h t = \frac{1}{2} \frac{J_h}{m_e} t \end{aligned}$$

In order to accurately predict these deflections, they have been simulated in Matlab (Mathworks) with numerical integration based on the trapezium rule to an accuracy of 0.1 mm for the spatial beam propagation and ${0.5}^{\circ }$ for the integration step size. The simulations show that the helical and summation approach both arrive at the same force for the respective direction, which was anticipated, given the chosen modelling approach. It also serves to verify the choices made to model the axial elongation of the solenoid. Further to this, the longitudinal force acting along the beam is found to be zero, which is in agreement with the Lorentz-force vector. On the other hand, the deflections occurring transversal to the beam are both expected due to the vector cross product $\vec {v} \times \vec {B}$ in the Lorentz-formula, as will be seen clearly in the field-based model in the next section. From this model it can also be deduced that the direction of deflection depends on the rotational direction of the current, which in the standard field model determines the magnetic field direction and thus the corresponding deflection. Here we can find a deflection of the beam in the positive y-direction and negative z-direction for mathematically positive rotating electrons and in the case of negative rotation, the deflection occurs in negative y- and positive z-direction. This is consistent for the expected vertical deflection direction from the right-hand-rule, respectively left-hand-rule in the standard field based approach, which will be examined next.

### Field model

For the calculation of the magnetic field a Matlab code by D. Cébron is used^28^ which is based on the work of Derby and Olbert^30^ and Callaghan and Maslen^29^. The latter is contained in a technical note from NASA, in order to calculate the fields inside and outside of any finite solenoid or magnet. This simulates the radial component, $B_{\rho }$, and the axial component, $B_z$, based on the residual field, $B_{res} = \mu _0 n_{unit} I$, with $n_{unit}$ being the number of turns per unit length, and then solving the complete elliptic integrals for the magnetic fields. These are^28^:

21 $$\begin{aligned} B_{\rho } = \frac{B_{res}}{\pi } \sqrt{\frac{R}{\rho }} \left[ \frac{\omega ^2_{\pm } - 2}{2 \omega _{\pm }} \mathcal {K} (\omega _{\pm }) + \frac{\mathcal {E} (\omega _{\pm })}{\omega _{\pm }} \right] ^+_- \end{aligned}$$

where $\mathcal {K}$ and $\mathcal {E}$ are the complete elliptic integrals of the first and second kind and

22 $$\begin{aligned} \omega _{\pm } = \sqrt{\frac{4R\rho }{(R + \rho )^2 + \xi ^2_{\pm }}} \end{aligned}$$

with $\xi _{\pm } = z \pm L/2$. Then the following definitions are made for the axial magnetic field,

23 $$\begin{aligned} \gamma= & {} (R - \rho )/(R+ \rho ), \end{aligned}$$

24 $$\begin{aligned} \zeta _{\pm }= & {} \sqrt{\frac{(R- \rho )^2 + \xi ^2_{\pm }}{(R+ \rho )^2 + \xi ^2_{\pm }}}, \end{aligned}$$

25 $$\begin{aligned} \chi _{\pm }= & {} \frac{\xi _{\pm }}{\sqrt{(R+\rho )^2 + \xi ^2_{\pm }}}. \end{aligned}$$

**Figure 2:**
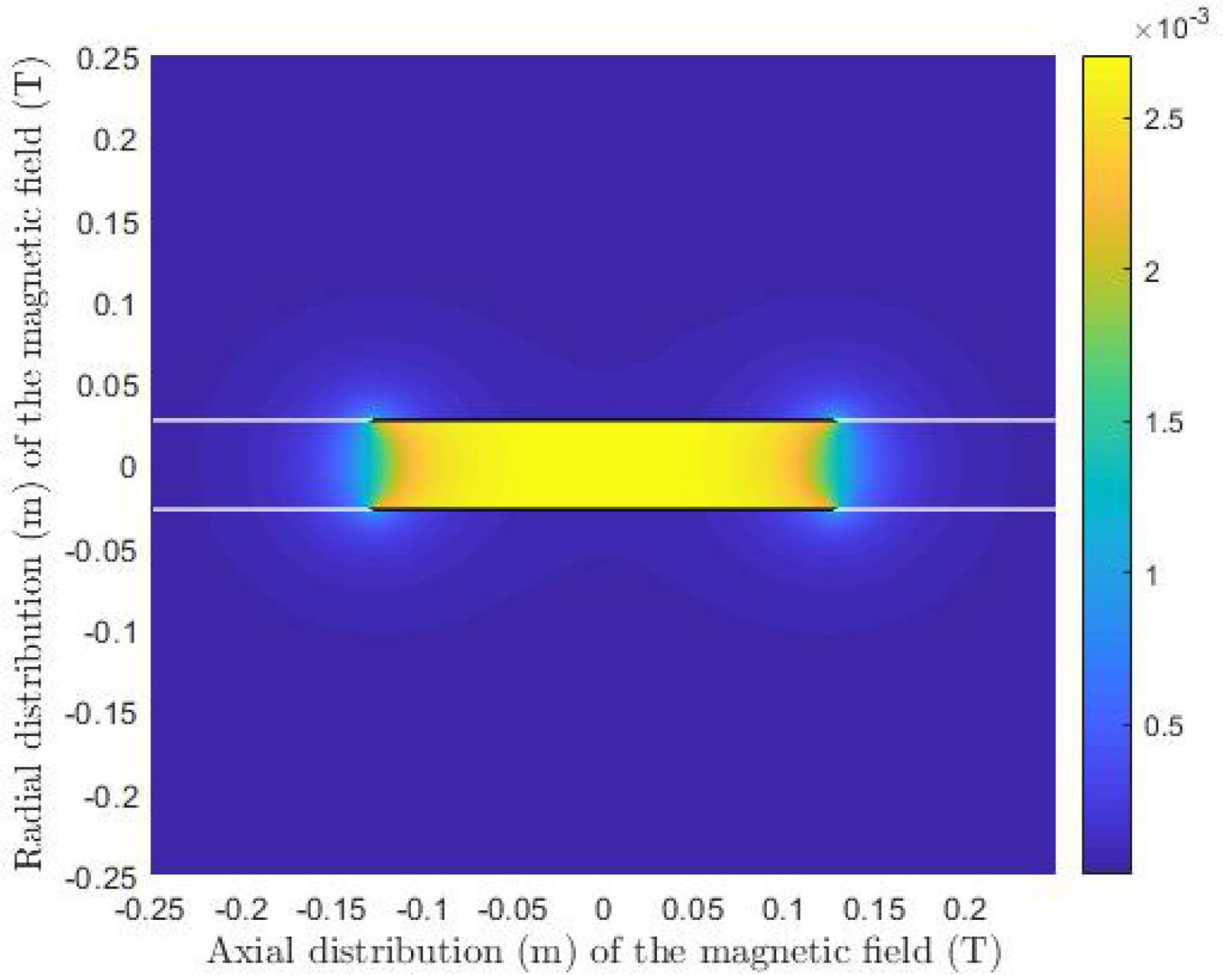
Field of solenoid S2 generated with the field calculations from^28^. The field strength (*T*) is shown around the solenoid, except for the white lines at the radial position $\rho =R$, as the algorithm did not converge at these points.

The axial field is then for $\zeta _+ < 1$

26 $$\begin{aligned} B_z = \frac{B_r}{\pi } \frac{R}{R+\rho } \frac{1}{\gamma + 1} \left[ \chi _{\pm } \left( \mathcal {K} \left( \sqrt{1-\zeta ^2_{\pm }} \right) + \gamma \Pi \left( 1-\gamma , \sqrt{1-\zeta ^2_{\pm }}\right) \right) \right] ^+_- \end{aligned}$$

and for $$\zeta _{\pm } \geqslant 1 $$

27 $$\begin{aligned} B_z = \frac{B_r}{\pi } \frac{R}{R+\rho } \frac{1}{\gamma (\gamma + 1)} \left[ \frac{\chi _{\pm }}{\zeta _{\pm }} \left( \gamma \mathcal {K} \left( \sqrt{1-\frac{1}{\zeta ^2_{\pm }}} \right) + \Pi \left( 1-\frac{1}{\gamma ^2} , \sqrt{1-\frac{1}{\zeta ^2_{\pm }}}\right) \right) \right] ^+_- \end{aligned}$$

with $\Pi$ being the complete elliptic integral of the third kind. The total calculated field strength with this approach can be seen in Fig. 2 which has been simulated with the same accuracy as in the ‘Weber model’ section. The white lines in the Figure, situated at $\pm R$, are where the algorithm used did not converge.

Next, to perform the vector cross product and deduce the deflection along the y and z axes, we convert the field to Cartesian coordinates at an angle of $\theta = \frac{3}{2} \pi$ as the beam passes below the solenoid. Thus

28 $$\begin{aligned} \vec {B} = \begin{pmatrix} 0 \\ - B_{\rho } \\ B_z \end{pmatrix} \end{aligned}$$

and we can get the deflecting force

29 $$\begin{aligned} \vec {F}_L = q( \vec {v}_1 \times \vec {B}). \end{aligned}$$

With that the Larmor radius can be obtained in the usual way, $r_L = \frac{m_e v_e^2}{F_L}$ and the deflection is obtained by using $r_L \pm \sqrt{r_L^2 - d^2}$, where *d* is the travel distance of the beam and the sign depends on the direction of deflection.

Apart from that, an impulse approach was also used to calculate the deflections, where the force acting on the beam has been calculated following the beam trajectory, starting from the position where the Larmor radius is obtained to the point where the beam is intercepted by the fluorescent screen. Thus, the local force is obtained with the same granularity as in the previously described model. The forces can then be integrated according to Eq. (18) and the final deflection is again obtained using Eq. (20). Both Larmor radius and impulse approach then give the same results for deflections in the y and z directions.

Additionally, the model by Farley and Price^53^ to roughly estimate the field of a finite solenoid has also been used to check the deflection at the position $z_1$ = 0 when the beam is centred across the coil. This model however, can only predict the vertical deflection at that specific point as it was not designed for inhomogeneous fields.

### Modelling with CPO

Finally, the deflections across the solenoid are also modelled with state-of-the-art commercially available software CPO^22^. Here the solenoid is modelled as a stack of current loops, which is one of the two standard methods available in the software. Following the geometries shown in the ‘Experimental setup’ section, the solenoid is set to elongate on the z-axis and with the coordinate origin aligning with the centre of the solenoid. A current of 1.00 A is supplied to rotate in the mathematically negative sense, which represents positive rotation of the electrons moving through the solenoid. The beam going across the solenoid is seen as individual rays of electrons and traced with the direct method. Both the databuilder file that is used to implement the experimental setup and the field that is generated by the software (see Fig. SF1) are given in the supplementary information. The simulation results to model electron beam deflection across a solenoid from these three models can be found in Tables 2 and 3 in the ‘Results’ section, where they are also compared to the experimental results.

## Experimental setup

**Figure 3:**
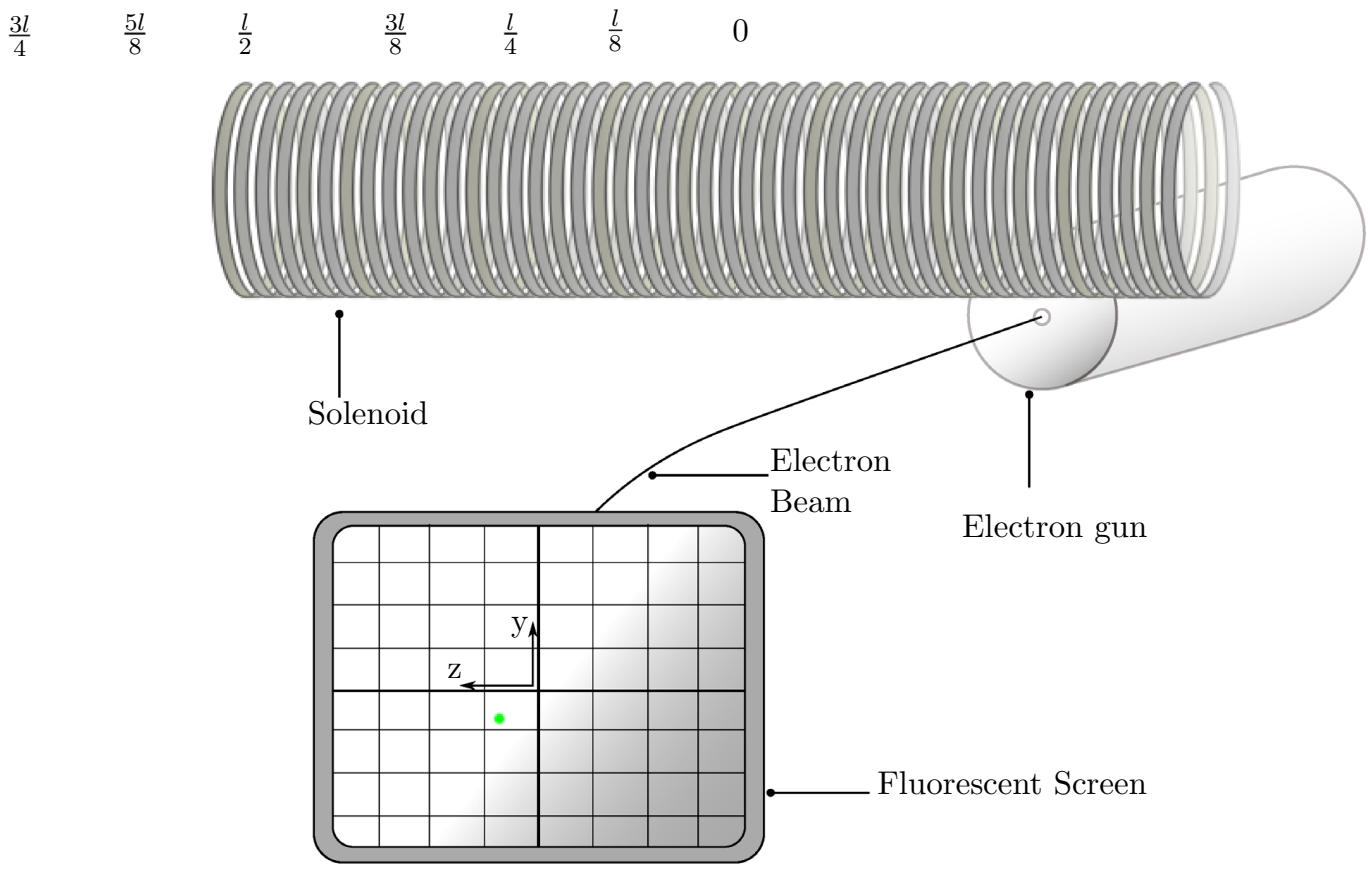
Solenoid positioned across the electron beam from the electron gun at $z_1 = 0$ where the electrons are deflected and intercepted by the fluorescent screen with an indicated beam spot (green). Also depicted are the positions $\frac{l}{8}$ to $\frac{3l}{4}$ where the beam can also be positioned with reference to the centre of the solenoid.

A sketch of the relatively simple experimental setup is shown in Fig. 3, depicting an electron beam (from an electron gun) which is deflected across the current carrying solenoid and then strikes a fluorescent screen. The electron gun and the fluorescent screen were adapted from a Hameg 203-6 cathode ray oscilloscope, so that the solenoid can be located across the electron beam, at right angles to the electron beam. The circuitry of the oscilloscope has been modified into a control unit that connects to the electron gun through an external cable.

The electron gun tube operates at an acceleration voltage of 2000V, which gives the electrons a terminal velocity of

30 $$\begin{aligned} v_e = \sqrt{\frac{2eV}{m_e}} \approx 2.65 \times 10^{7}\,\hbox {m s}^{-1} \end{aligned}$$

after passing the anode, at a distance of 7 cm from emission. Propagating in a straight line the fluorescent screen is then intercepted after another 21.5 cm of beam travel. The glass evacuated tube enclosing the electron gun has a diameter of 50 mm and the solenoid sits on top of it, at a distance of 3 cm from the anode. For our choice of coordinates, the beam propagates at a height $h = -(25+R)\,\hbox {mm}$ and travels 18.5 cm from the coordinate origin to the fluorescent screen with this arrangement.

The phosphor screen has a graticule divided in 10 mm steps, with subdivisions of 2 mm that give a rough indication of where the beam is intercepted by the screen after the deflection. The spot left by the beam can be focused to a size of roughly 1 mm in diameter and is indicated by the green coloured spot in Fig 3. To deflect the beam, three different solenoids are used: length 25 mm (S1), length 255 mm (S2) and length 500 mm (S3), with different radii and numbers of windings, as documented in Table 1. By definition, the shortest solenoid with 25 mm length can be considered as a ‘coil’, but will be further referred to as solenoid S1. A continuous DC-current of 1.00 A is supplied to the solenoid with a benchtop power supply.

**Table 1:** Properties of the three Solenoids S1, S2 and S3 that are used in the experiments.

Solenoid	Radius (mm)	Length (mm)	Absolute number of windings and [$n_{unit}$]	Current (A)
S1	20	25	61 [2440]	1.00
S2	27	255	560 [2200]	1.00
S3	30	500	1300 [2600]	1.00

The solenoids are set in such a way that the beam is deflected at different positions relative to the coordinate origin, so that the beam crosses different regions of the field, which are getting increasingly more fringing closer to the solenoid ends. For the shortest solenoid, it is first set for the beam to cross at $$z_1 = 0$$, per se at the middle of the solenoid length and then moved in steps of one quarter of the length towards the edge of the solenoid, so that it passes at $$z_1 =\frac{l}{4} $$ and then at $$z_1 =\frac{l}{2} $$ where it is aligned with the end of the solenoid on the z-axis. The solenoid is then moved for the beam to be positioned at $$z_1 =\frac{3l}{4} $$ and $$z_1 = l $$, while the solenoid is kept at the same height. At these points the beam is not crossing the coil directly any more, as it sits at a distance further from the origin than the solenoid’s axial elongation. These positions are also implied in Fig. 3 for a visual representation of where the beam is travelling in relation to the solenoid origin.

For the two longer solenoids, S2 and S3, the beam is set in $\frac{1}{8}$ steps of the solenoid length from the centre position, $z_1 = 0$, up to the point $z_1 =\frac{3l}{4}$ which are again shown in Fig. 3. Especially at the positions around $z_1 =\frac{l}{2}$, it is said that the field is fringing and strongly deviates from the ideal homogeneous field associated with an infinite solenoid. Furthermore, the shortest coil, S1, will have a very inhomogeneous field according to theory due to its length and radius being almost the same, with a relatively small number of windings.

To obtain measurement of beam deflections the following procedure is adopted. The solenoid is located at a given position across the e beam which is then focused and centred in the middle of the screen. The solenoid current is now switched on and adjusted to provide a magnitude of 1.00 A; the beam deflection is noted. The deflection on the screen is acquired with a plastic vernier along with the help of the graticule on the screen; immediately after the power supply is switched off. The procedure is repeated 3 times in order to acquire a mean deflection for the beam spot.

## Results and discussion

**Figure 4:**
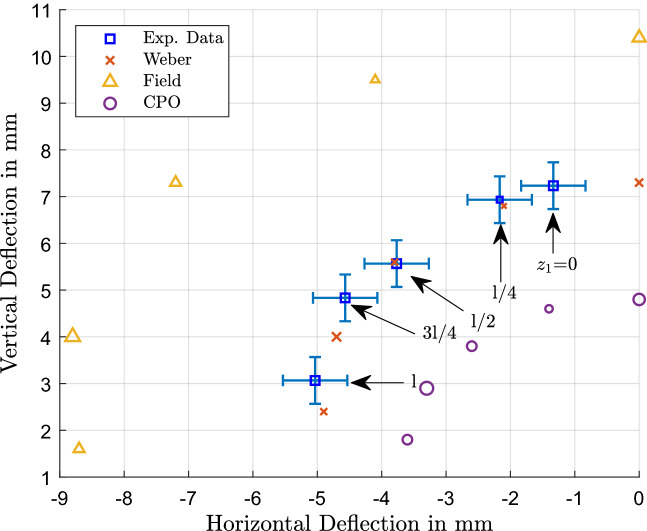
Measured and predicted deflections of the electron beam across S1 for both vertical and horizontal directions. In the bubble chart, the blue squares show the experimental data and are labelled with the beam position relative to the solenoid centre, i.e. the axial or “z”-position (as illustrated in Figure 3). For each given beam position, the size of the data points relating to that position are uniquely the same size, so that predictions from the Weber model (red x), Field model (yellow triangle) and CPO (purple circle) all have the same size marker (as the corresponding blue square/experimental measurement) for each given “z” position.

**Figure 5:**
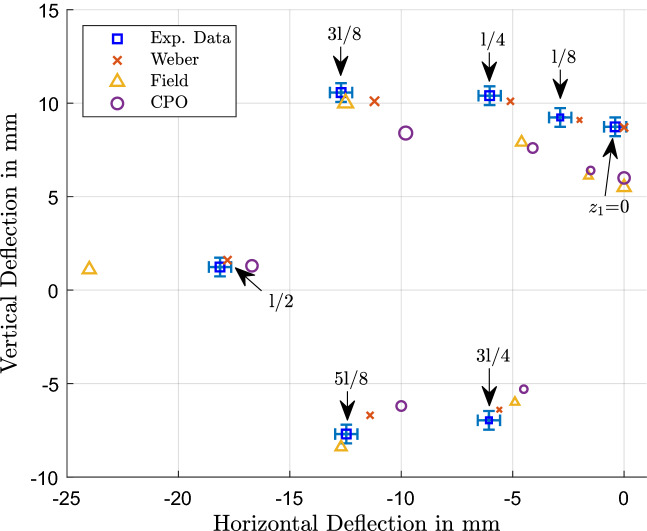
Measured and predicted deflections of the electron beam across S2 for both vertical and horizontal directions. The blue squares show the experimental data, the red x (Weber model), yellow triangle (Field Model) and purple circle (CPO) show the predictions of the various models whereby all data points of the same, unique size relate to a given axial (i.e., “z”) beam position.

**Figure 6:**
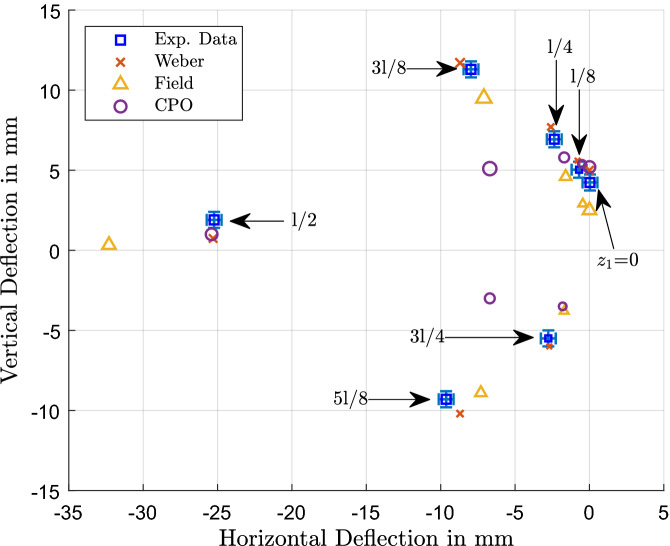
Measured and predicted deflections of the electron beam across S3 for both vertical and horizontal directions. The blue squares show the experimental data, the red x (Weber model), yellow triangle (Field model) and purple circle (CPO) show the predictions of the various models whereby all data points of the same, unique size relate to a given axial (i.e., “z”) beam position.

The beam deflections measured in the vertical and horizontal directions across the three solenoids are summarised in Figs. 4, 5 and 6. Figure 4 shows the measured deflection in the y and z direction along with the predictions from the different models for solenoid S1; similarly, Fig. 5 shows S2 and Fig. 6 shows S3. The experimental data is shown in blue squares for each of the beam positions and has corresponding predictions from the Weber-type model (red x), the field based model (yellow triangle) and numerical field model using CPO (purple circle). The data points of the measurements are labelled with the respective beam positions on the z-axis where the solenoid was crossed, starting from $l=0$. The size of the four data points for a specific position are the same such that the predictions can be related to the relevant experimental data point for a given position.

It can be seen very clearly in Fig. 4 that the overall trend and deflection positions for the inhomogeneous field of the short solenoid S1 are met well with the theoretical predictions from the Weber model. By contrast, calculations based on the field model show a greater departure from measurement, as it overestimates the deflection. The results from CPO are closer to the experimental values than the analytical field model, but the deflection values are underestimated in this case.

Similarly, as indicated in Fig. 5, deflections across solenoid S2 tend to be consistent with all three models; note the change in sign for the vertical deflection once the beam has been moved past the edge of the solenoid, where $z_1 > \frac{l}{2}$. Except for positions 3*l*/8 and 5*l*/8 where field theory predicts close agreement with measurements, the Weber-based model predicts deflections closest to the experimental values and CPO tends to underestimate the displacement. However, for the fringing field region at *l*/2 the field model greatly overestimates the horizontal deflection, whereas the direct model provides an accurate prediction and CPO also gives a close estimate.

Figure 6 shows the data for the longest solenoid S3; a similar behaviour at *l*/2 is observed with the field model. Again it overestimates the horizontal deflection by a noticeable margin compared to the direct action prediction and CPO, which give a much closer result. Looking at the positions 3*l*/8 and 5*l*/8, the deflections are underestimated by the field model and CPO alike, while the direct action model is closest to the experimental measurements. Still the general trend of deflections with the sign change in vertical deflection past the edge of the solenoid is followed by all three of the models.

For the non-fringing region at $z_1 = 0$ to *l*/4 the vertical deflection is underestimated for the two longer solenoids and overestimated for S1 in the field-model, whereas CPO seems to generally underestimate the deflections. The predictions of the Weber model are closer to the experimental values in most cases. This is in agreement with the earlier work on this topic^37^ where a uniform field was assumed for the vertical deflection according to the model of Farley and Price^53^.

It is interesting to note that in the field-based model, the part of the magnetic field responsible for the vertical deflection is the axial component $B_z$, whereas the radial component $B_\rho$, respectively $B_y$ in Cartesian coordinates, is responsible for the horizontal deflection, due to the vectorial cross product in the Lorentz force formula. In contrast, the direct-action model according to Weber predicts the deflection by the respective force component along that direction, the y-component of the force is thus responsible for the vertical deflection and the z-component is responsible for the horizontal deflection. It might well be possible to relate the Weber force components to the magnetic field strength in the future. Despite that conceptual difference, all three models can predict the deflection within a certain accuracy, although our investigation shows that, for this specific setup, the direct-action model seems to deliver the most accurate results.

For complete comparison, Tables 2 and 3 show the mean of the experimental data and the simulation results for each of the models. For the vertical deflection at $l = 0$ the Farley and Price model (F&P) is also included for consistency, but this model cannot predict the other deflections.

**Table 2:** Measured and predicted values for the vertical deflection $y_d$ across all three solenoids.

Solenoid	$z_1$	Vertical deflection $y_d$				F&P
		Experimental	Weber model	Field model	CPO	
S1	0	7.2	7.3 (98.6%)	10.4 (55.5%)	4.8 (66.7%)	N/A
	*l*/4	6.9	6.8 (98.5%)	9.5 (62.3%)	4.6 (66.7%)	
	*l*/2	5.6	5.6 (100%)	7.3 (76.7%)	3.8 (67.8%)	
	3*l*/4	4.8	4.0 (89%)	4.0 (80%)	2.9 (60.4%)	
	*l*	3.1	2.4 (77.4%)	1.6 (51.6%)	1.8 (58.1%)	
S2	0	8.7	8.7 (100%)	5.5 (63.2%)	6.0 (69%)	7.0
	*l*/8	9.2	9.1 (98.9%)	6.1 (66.3%)	6.4 (69.6%)	
	*l*/4	10.4	10.1 (97.1%)	7.9 (76%)	7.6 (73.1%)	
	3*l*/8	10.6	10.1 (95.3%)	10.0 (94.3%)	8.4 (79.2%)	
	*l*/2	1.2	1.6 (66.7%)	1.1 (91.7%)	1.3 (91.7%)	
	5*l*/8	− 7.7	− 6.7 (87%)	− 8.4 (90.9%)	− 6.2 (80.5%)	
	3*l*/4	− 7.0	− 6.4 (91.4%)	− 6.0 (85.7%)	− 5.3 (75.7%)	
S3	0	4.2	5.0 (81%)	2.5 (59.5%)	5.2 (76.2%)	2.7
	*l*/8	5.0	5.6 (88%)	2.9 (58%)	5.4 (92%)	
	*l*/4	6.9	7.7 (88.4%)	4.6 (66.7%)	5.8 (84.1%)	
	3*l*/8	11.3	11.7 (96.5%)	9.5 (84.1%)	5.1 (45.1%)	
	*l*/2	1.9	0.7 (36.8%)	0.3 (15.8%)	1.0 (52.6%)	
	5*l*/8	− 9.3	− 10.2 (90.3%)	− 8.9 (95.7%)	− 3.0 (32.3%)	
	3*l*/4	− 5.5	− 6.0 (90.9%)	− 3.8 (69.1%)	− 3.5 (63.6%)

**Table 3:** Measured and predicted values for the horizontal deflection $z_d$ across all three solenoids.

Solenoid	$z_1$	Horizontal Deflection $z_d$			
		Experimental	Weber model	Field model	CPO
S1	0	− 1.3	0	0	0
	*l*/4	− 2.2	− 2.1 (95.5%)	− 4.1 (13.6%)	− 1.4 (63.6%)
	*l*/2	− 3.8	− 3.8 (100%)	− 7.2 (10.5%)	− 2.6 (68.4%)
	3*l*/4	− 4.6	− 4.7 (97.8%)	− 8.8 (8.7%)	− 3.3 (71.7%)
	*l*	− 5.0	− 4.9 (98%)	− 8.7 (26%)	− 3.6 (72%)
S2	0	− 0.4	0	0	0
	*l*/8	− 2.9	− 2.0 (69%)	− 1.6 (55.2%)	− 1.5 (51.7%)
	*l*/4	− 6.0	− 5.1 (85%)	− 4.6 (76.7%)	− 4.1 (68.3%)
	3*l*/8	− 12.7	− 11.2 (88.2%)	− 12.5 (98.4%)	− 9.8 (77.2%)
	*l*/2	− 18.1	− 17.8 (98.3%)	− 24.0 (67.4%)	− 16.7 (92.2%)
	5*l*/8	− 12.5	− 11.4 (91.2%)	− 12.7 (98.4%)	− 10.0 (80%)
	3*l*/4	− 6.1	− 5.6 (91.8%)	− 4.9 (80.3%)	− 4.5 (73.8%)
S3	0	0.0	0	0	0
	*l*/8	− 0.7	− 0.7 (100%)	− 0.4 (57.1%)	− 0.5 (71.4%)
	*l*/4	− 2.4	− 2.6 (91.7%)	− 1.6 (66.7%)	− 1.7 (70.8%)
	3*l*/8	− 8.0	− 8.7 (91.3%)	− 7.1 (88.8%)	− 6.7 (83.8%)
	*l*/2	− 25.2	− 25.3 (99.6%)	− 32.2 (72.2%)	− 25.4 (99.2%)
	5*l*/8	− 9.6	− 8.7 (90.6%)	− 7.3 (76%)	− 6.7 (69.8%)
	3*l*/4	− 2.8	− 2.7 (96.4%)	− 1.7 (60.7%)	− 1.8 (64.3%)

It can be seen from these Tables that in the F&P model the electron beam does not reach the fluorescent screen after being deflected by the shortest solenoid, because the produced field deflects the beam too much in this model. Also it is apparent from the data that the beam is deflected to the opposite vertical direction for S2 and S3 at the points 5*l*/8 and 3*l*/4 and each model correctly predicts the change in sign.

The Table only shows the experimental and simulation data for mathematically positive rotating electrons (which is negative rotation in terms of conventional current flow); changing the direction of current flow will invert the deflection directions, in the experiments as well as in the models. So the three models, respectively both field theory and direct-action-at-a-distance theory agree on the same trend that is seen for a deflection in the fringing fields of finite solenoids. The relative accuracy of the compared models can be obtained according to Eq. (31):

31 $$\begin{aligned} \text {Accuracy} = 1- \frac{|\text {Modelled Deflection}-\text {Measured Deflection}|}{\text {Measured Deflection}} \end{aligned}$$

Overall, the field model is found to have an average accuracy of 67.4%, followed by CPO with 73.2% and the Weber model with 91.7 % accuracy for the modelled deflections relative to the experimental values.

Further to the results shown here, the deflections are shown for the individual vertical and horizontal deflections across each solenoid in the supplementary information (in Figures SF2–SF10), which also includes the total deflection $\zeta = \sqrt{y_d^2 + z_d^2}$ depicting the polar equivalent of deflection magnitude.

## Conclusion

The deflection of an electron beam across three differently sized solenoids has been tested experimentally and was predicted with three distinct models. One is a field theory model based on the equations of Maxwell and Lorentz, the second is a field based numerical model, from a commercial software package, which utilises the boundary element method and the third is a direct-action-at-a-distance-theory based on the Weber force.

It was found that, in general, for the limiting condition $v \ll c$ in the low velocity regime, the experimental data has good agreement with the Weber-based model. Especially for the shortest solenoid S1 (25 mm length) where the field is non-uniform, the field model is significantly overestimating the deflection and, to a lesser extent, the numerical field model is underestimating. For the two longer solenoids, S2 (255 mm) and S3 (500 mm), where the field is slightly more uniform, the direct-action model produces overall the most accurate predictions. However, all models follow the observed trend and overall the Weber model is found to be 91.7% accurate relative to the experimental data.

The Weber theory is based on minimal assumptions compared to the field approach, as it does not directly involve the calculation of field entities $\vec {B}$ and $\vec {E}$, nor leakage flux or vector potential. It offers the more accurate predictions, for this particular case, by directly calculating the forces involved. This makes it especially interesting from an engineering point-of-view, as it bypasses the calculation of the field and provides the electromagnetic force directly.

The present investigation raises of course the question for the behaviour of charged particles in the higher velocity regimes, when the movement is approaching the speed of light, where the field model with the Lorentz-correction factor is experimentally proven to deliver accurate results. The problems with the direct-action theory in this case have been discussed^54^ and attempts have been made to amend the theory^55^ to reflect better the behaviour of fast moving charges and the matter is still subject to recent investigation^38^. Nevertheless this shows that both theories hold certain benefits and drawbacks that can be used advantageously depending on the situation as we consider that both approaches complement each other. As we are limiting the present investigation to the low velocity regime, the results could be relevant for several applications in the field of charged particle dynamics, not limited to charge guidance by solenoids.

## Electronic supplementary material

Supplementary material 1
