# Supplementary material for: Accurately predicting electron beam deflections in fringing fields of a solenoid
Source: Sci Rep. 2020 Jul 2;10:10903. doi: 10.1038/s41598-020-67596-0 (PMC7331722; doi:10.1038/s41598-020-67596-0)
Supplement: Supplementary file 1 — Supplementary material 1 [file 41598_2020_67596_MOESM1_ESM.pdf]

Supplementary Information

Accurately Predicting Electron Beam Deflections in Fringing Fields of a Solenoid

Christof Baumgärtel[1], Ray T. Smith[1], and Simon Maher[1,*]

[1] *Department of Electrical Engineering and Electronics, University of Liverpool*
[*] *corresponding author, s.maher@liverpool.ac.uk*

April 16, 2020

CPO Databuilder file for solenoid S2

```
CPO3D -based on 4th test, relativistic cyclotron motion
tempa.dat name of hidden output file, for processed data
tempb.dat  name of main ray output file, for ray data
n      v  n/p/m/a for print level, cumulative, colour electrodes
0 0 0 0   voltage reflection symmetries in x,y,z,x=y planes
1     number of different voltages (time-independent)
0.001  5    0   allowed consistency error, side/length ratio check, allow outside zs
n  apply inscribing correction (a/s/n=always/sometimes/never)
cylindrical electrode
1 1000 1000 1000  radius, centre of 1st end
1001 1001 1001  centre of 2nd end
1  1  numbers of 2 applied voltages (can be same)
1    5       total nr of subdivs and 0,   or subdivs along and around axis
colour    1
end of electrode information
0     1  0.5    final nmbr segs, nmbr steps, weight
1e-07               charge inacc,non-0 total Q,improve matrix,import,sp-ch
end of segment information
0.0000000E+00       rods at +/- x               applied voltages
s    solenoid, type 1
1 27 27 560    current, radii, nr of turns
0 0 127.5 0 0 -127.5    coordinates of 2 ends of solenoid
n      no more magnetic fields from menu
0 0 0 0 c  symmetries of rays in yz,zx,xy and x=y planes, compounded transformation
n n n        no more potentials and fields along a line
start of ray information
d      direct (d), or mesh (m) method and mesh spacing
p    'n/p/m/a' for 'nearlyzero/partial/most/all' printing level, rho/radius
-250 250    minimum and maximum x(mm) of screen 2D fields of view
-250 250    minimum and maximum y
-250 250    minimum and maximum z
-250 250     minimum and maximum x(mm) of rays
-250 250     minimum and maximum y
```

```
-250 250     minimum and maximum z
-1e+10 1e+10    minimum and maximum vx(m/s) of rays
-1e+10 1e+10    minimum and maximum vy
-1e+10 1e+10    minimum and maximum vz
+ y   direction of time, stop when ray first hits an electrode
1e+10   final time (ms)
1           10          =max step length,interp pts
-0.0001 0.0001  fractional inaccuracies for (1) ray tracing and (2) potentials and fields
1              nr test planes,mult cross,iter foc,phase spc,scatt,quant,stp_tst
1 0 0 185      a,b,c,d of test plane defined by a*x+b*y+c*z=d
el       'el' for electron; or 'co' or 'va' for other particles
k kinetic energy (k); or total energy (t) and potential
set of single rays:
-30 -52 0 1 0 0 2000 0            x,y,z,vx,vy,vz,eV,I
last of this set of rays
n     calculate space-charges?
```

Supporting Figures

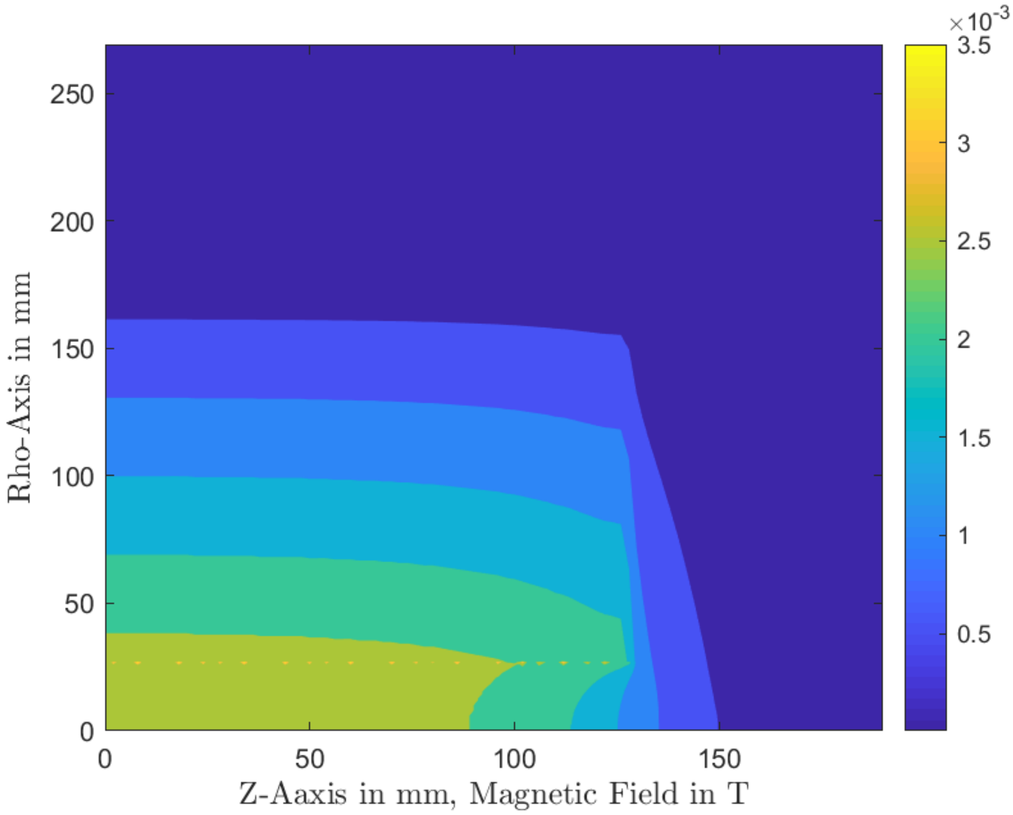

Figure SF1: Magnetic field of solenoid S2 that is generated by CPO, extracted with a 2 mm granularity. Due to the symmetry of the field only positive ρ- and z-axis are shown.

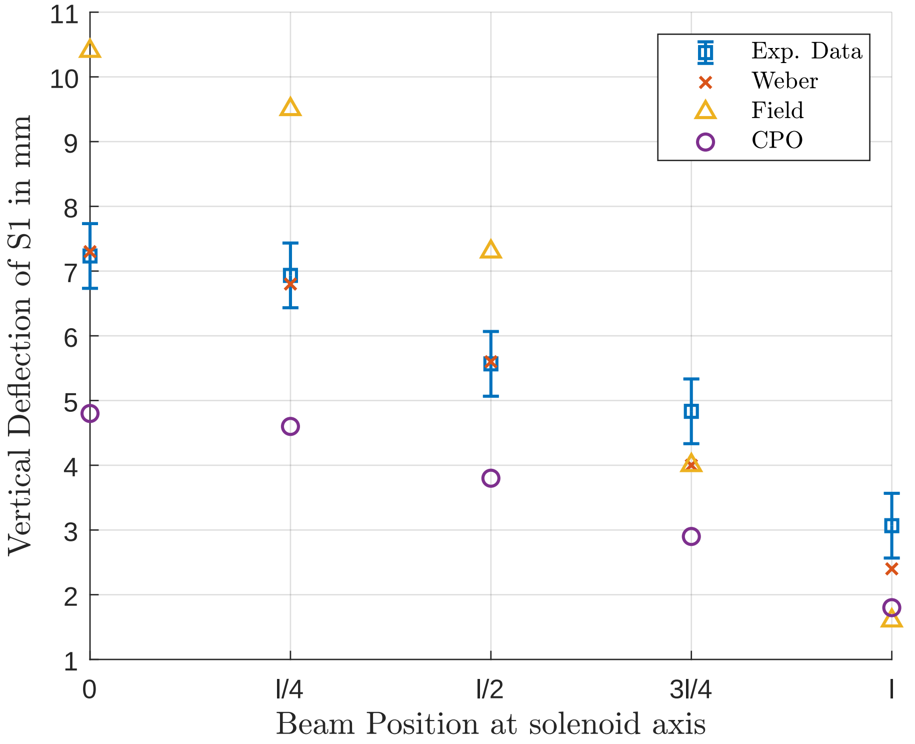

Figure SF2: Vertical deflection y_d of the electron beam across solenoid S1

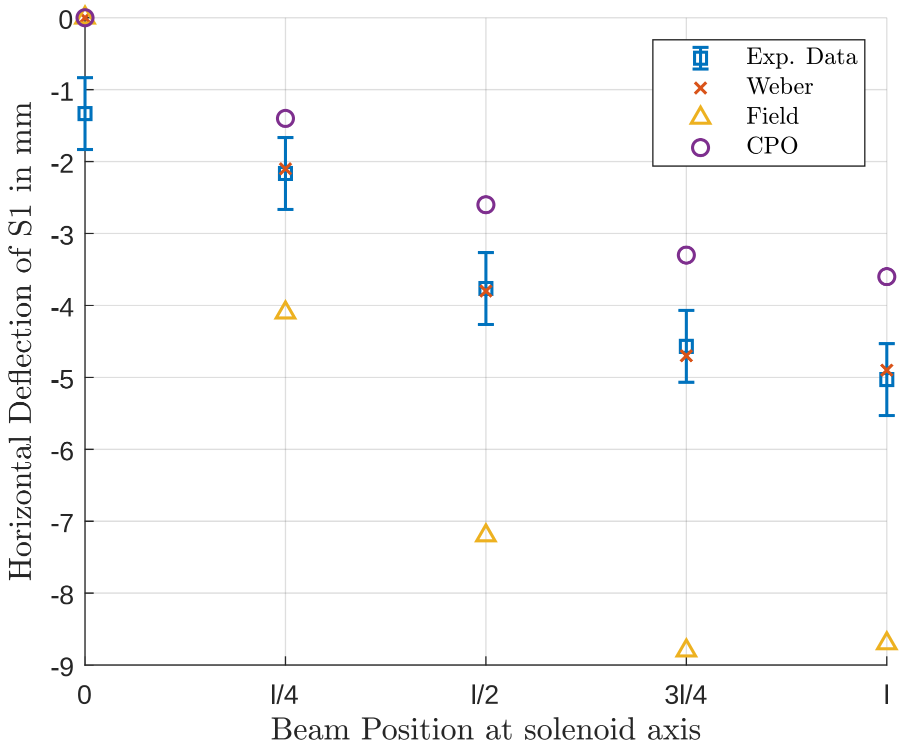

Figure SF3: Horizontal deflection z_d of the electron beam across solenoid S1

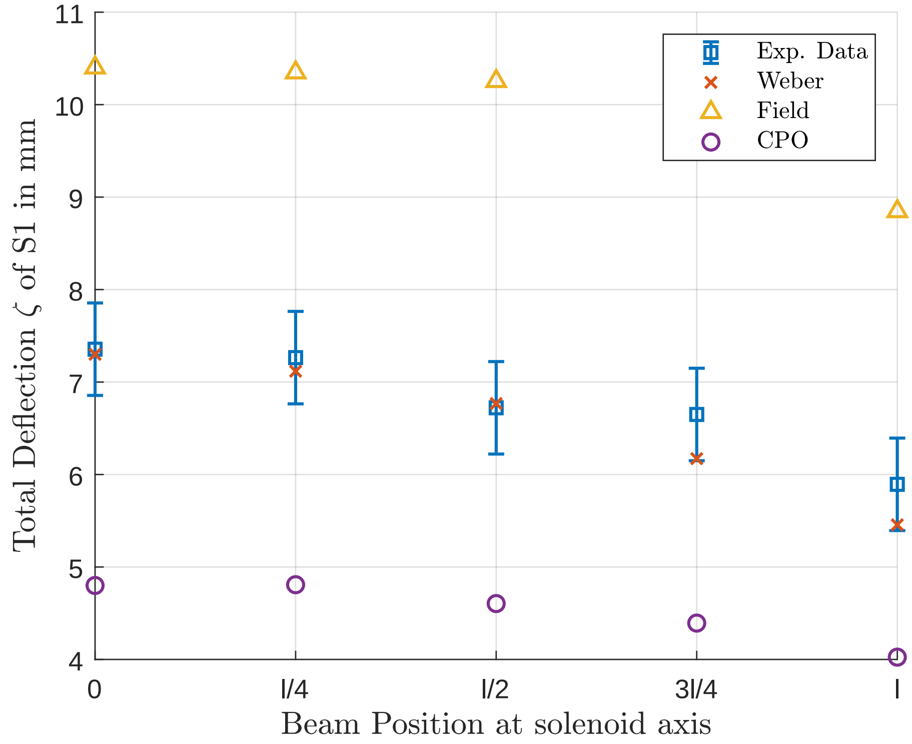

Figure SF4: Total deflection $\zeta = \sqrt{y_d^2 + z_d^2}$ of the electron beam across solenoid S1

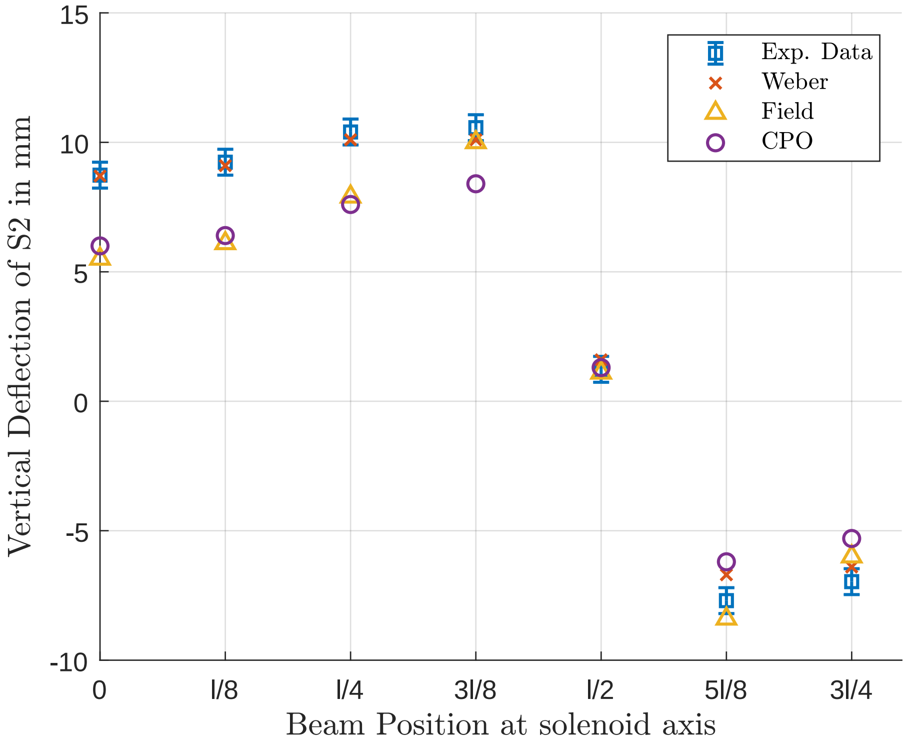

Figure SF5: Vertical deflection y_d of the electron beam across solenoid S2

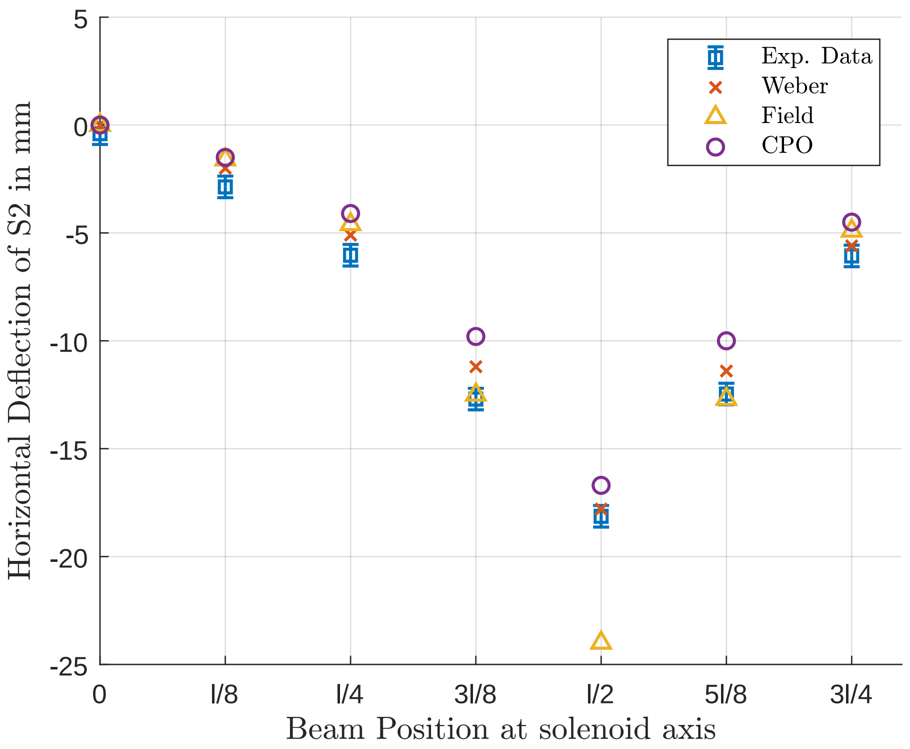

Figure SF6: Horizontal deflection z_d of the electron beam across solenoid S2

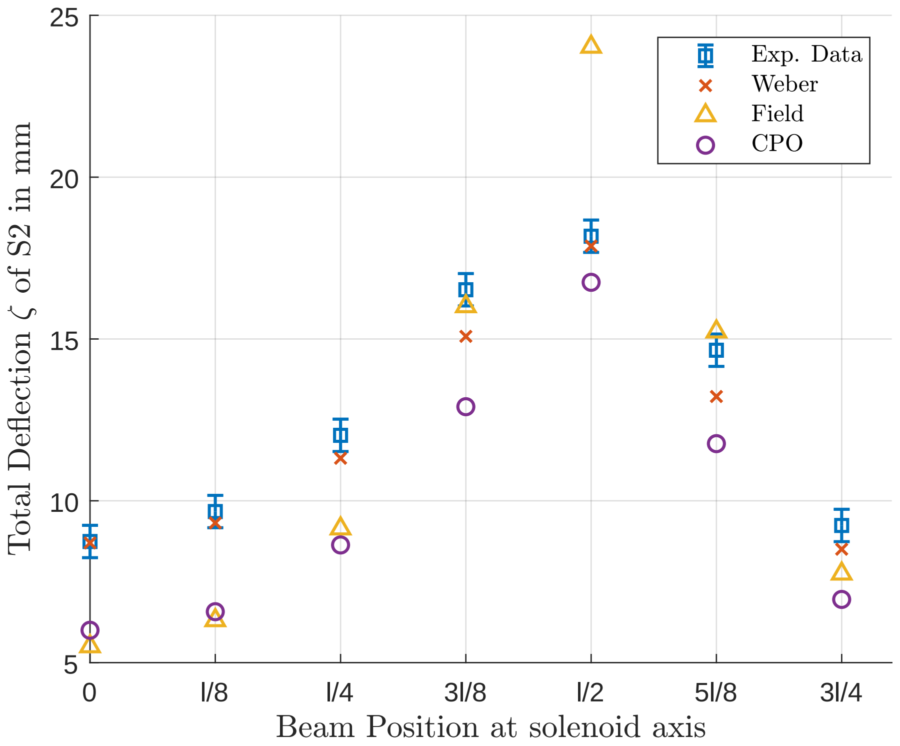

Figure SF7: Total deflection $\zeta = \sqrt{y_d^2 + z_d^2}$ of the electron beam across solenoid S2

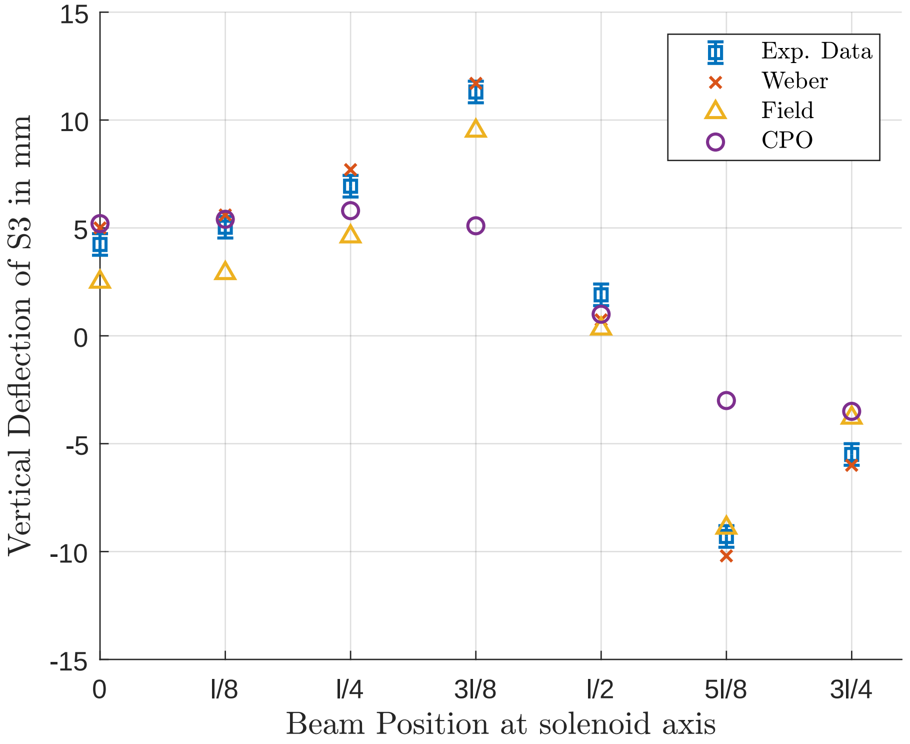

Figure SF8: Vertical deflection y_d of the electron beam across solenoid S3

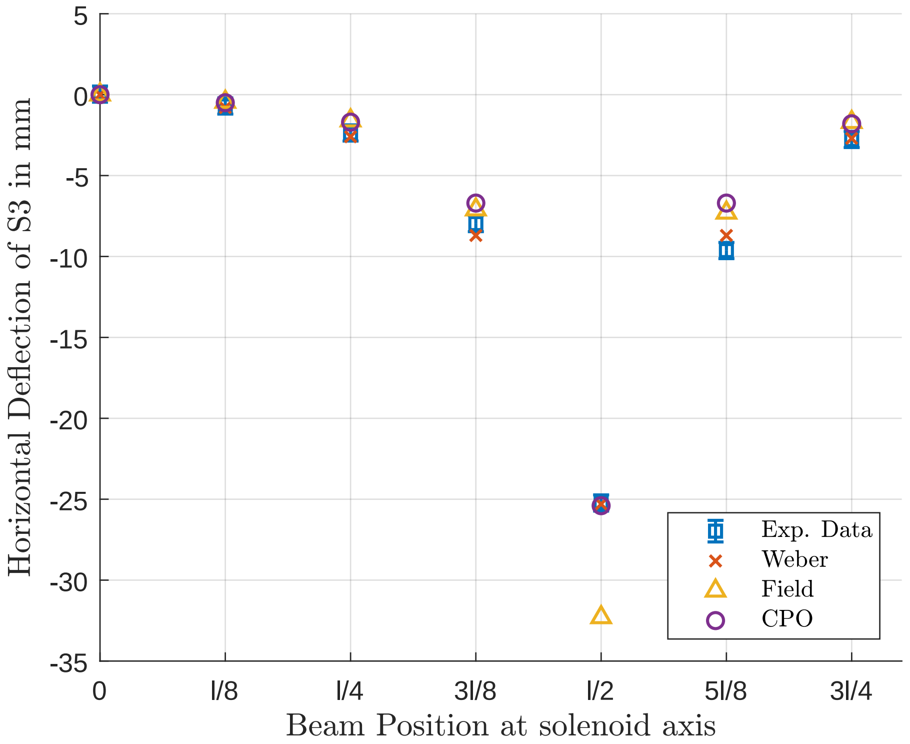

Figure SF9: Horizontal deflection z_d of the electron beam across solenoid S2

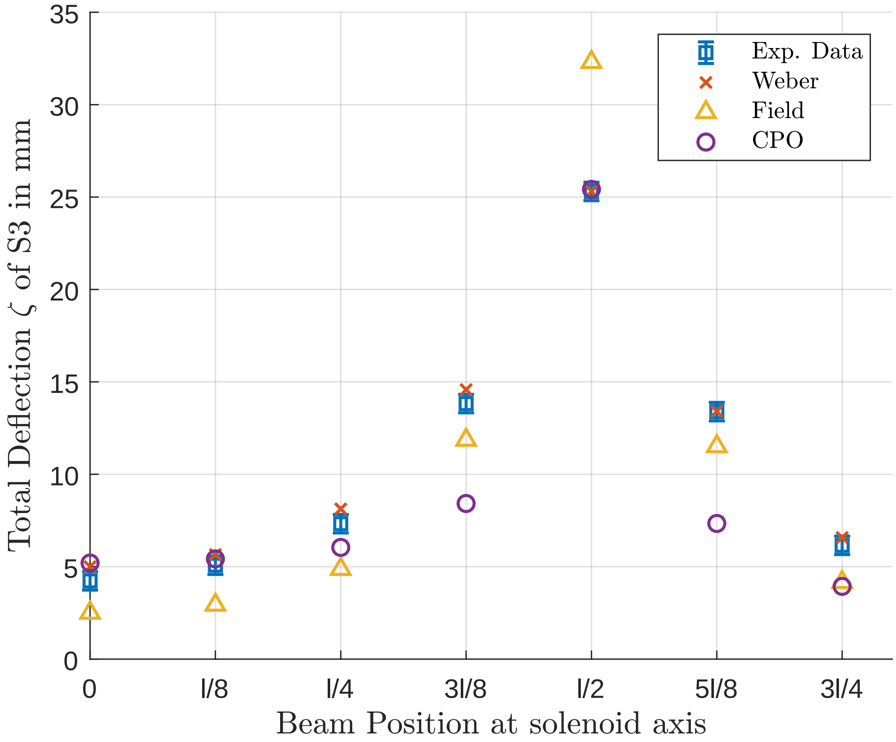

Figure SF10: Total deflection $\zeta = \sqrt{y_d^2 + z_d^2}$ of the electron beam across solenoid S3
